# Using reflectometry to minimize the dependence of fluorescence intensity on optical absorption and scattering

**DOI:** 10.1364/BOE.496599

**Published:** 2023-09-27

**Authors:** Augusto Arias, Maria Anastasopoulou, Dimitris Gorpas, Vasilis Ntziachristos

**Affiliations:** 1 Chair of Biological Imaging at the Central Institute for Translational Cancer Research (TranslaTUM), School of Medicine, Technical University of Munich, Munich, 81675, Germany; 2 Institute of Biological and Medical Imaging, Helmholtz Zentrum München, Neuherberg, 85764, Germany; 3 DZHK (German Centre for Cardiovascular Research), partner site Munich Heart Alliance, Munich, 81675, Germany

## Abstract

The total diffuse reflectance *R*_*T*_ and the effective attenuation coefficient *µ*_*eff*_ of an optically diffuse medium map uniquely onto its absorption and reduced scattering coefficients. Using this premise, we developed a methodology where *R*_*T*_ and the slope of the logarithmic spatially resolved reflectance, a quantity related to *µ*_*eff*_, are the inputs of a look-up table to correct the dependence of fluorescent signals on the media’s optical properties. This methodology does not require an estimation of the medium’s optical property, avoiding elaborate simulations and their errors to offer accurate and fast corrections. The experimental demonstration of our method yielded a mean relative error in fluorophore concentrations of less than 4% over a wide range of optical property variations. We discuss how the method developed can be employed to improve image fidelity and fluorochrome quantification in fluorescence molecular imaging clinical applications.

## 1. Introduction

Fluorescence molecular imaging (FMI) is increasingly being considered for guiding surgery [1] and diagnostic endoscopy [2] since it potentially allows for more sensitive and specific detection of disease over human vision or white light endoscopy methods. FMI uses fluorescent agents that are administered to tissue and can target and allow visualization of aspects of disease such as upregulated receptors or dysregulated enzymatic activity in cancer cells [3]. The fundamental premise of this technique is that the recorded fluorescence image represents the spatial bio-distribution of the fluorescent agent in tissue (i.e., the fluorophore concentration). However, this premise does not generally hold true, since the recorded fluorescence intensity (FI) depends not only on the fluorochrome concentration but also on the optical properties of the tissue imaged [4]. This dependence modulates the FI and may lead to erroneous readings, i.e. false positives and negatives.

During the last few years, the application of fluorescence lifetime for quantitative FMI has gained attention [5]. However, the lack of high-resolution imaging sensors, the system complexity, and the strong influence of signal-to-noise ratio on the lifetime quantification and spectral unmixing still make these approaches less appealing than the steady-state techniques. Therefore, this paper is focused on methods to correct the effects of varying tissue optical properties on fluorescence images based on steady-state measurements. The accuracy of those methods increases with their complexity, as reviewed as follows.

The simplest empirical method obtains the ratio between the raw fluorescence and the diffuse reflectance at the excitation wavelength [6] (‘F/R method’). Other formulations also include the ratio by the reflectance at the emission wavelength [7,8]. However, these corrections are more effective against variations in the optical absorption properties of tissues than variations due to scattering [9]. Other empirical techniques use simple calculations on wide-field fluorescence images at different spectral windows to minimize the effects of optical properties and autofluorescence [10]; for example, using the ratio of FIs from targeted tracers to untargeted tracers for fluorescence correction [11–13]. Although these approaches may be used to correct for depth, they are still prone to inaccuracies due the spatial and spectral variation of tissue optical properties. In addition, these approaches require dual labelling, which may decrease the brightness of the agent in each emission window.

To address the limitations of empirical methods, approaches based on determining the spatial variation of optical properties in tissues have been also considered [14–20]. In these approaches, the absorption ( $\mu_{a}$ ) and reduced scattering ( $\mu_{s}^{\prime }$ ) coefficients of tissue are estimated by experimentally collecting varying forms of information (including measuring the spectral diffuse reflectance [14,18,19,21,22], the spatially resolved diffuse reflectance [17] or the spatial modulation transfer function [22–27]) to be fitted with numerical or analytically-derived models of photon-propagation in tissue-like media with $\mu_{a}$ and $\mu_{s}^{\prime }$ values. While developing numerical models requires running highly computationally demanding simulations (e.g., Monte-Carlo (MC) algorithms), the analytical models are generally supported by simplifications on the experimental conditions. The computed $\mu_{a}$ and $\mu_{s}^{\prime }$ are then employed in numerical [18,20] or analytical [14–16,19] models of fluorescence photon propagation to modify the fluorescence intensity and estimate the spatial distribution of fluorescent agent concentration. Theoretical approximations, measurement errors and, importantly, errors associated with the ill-posed nature of computing tissue optical properties from experimental measurements [28] may reduce the accuracy of determining the spatially-dependent $\mu_{a}$ and $\mu_{s}^{\prime }$ , and these errors are propagated to the estimation of fluorescence agent concentration. Additionally, these methods are computationally time-intensive and may delay the overall FMI procedure. Therefore, there is a need for an accurate but time-efficient method to determine fluorophore distribution independent of the spatial variation of the tissue’s optical properties.

Aiming to offer a method with improved robustness against errors, we propose a novel model-independent approach where two reflectometry measurements obtained from the tissue are the inputs in a look-up table (LUT) that provides a factor for the optical property correction of FI. To achieve this, our method employs two images obtained after illuminating tissue with two disks (one large and one small). While the image of the large disk is processed to calculate the total diffuse reflectance ( $R_{T}$ ), the image of the small disk is used to calculate the spatially resolved reflectance (SRR, i.e. the profile of photon distribution in tissue as it appears on the surface). The SRR is processed by computing the slope of its logarithm ( $slope_{logSRR}$ ), which is nearly proportional to the effective attenuation coefficient ( $\mu_{eff}=\sqrt{3\mu_{a}\mu_{t}^{\prime }}$ , while $\mu_{t}^{\prime }=\mu_{s}^{\prime }+\mu_{a}$ ) [29]. Since $R_{T}$ and $\mu_{eff}$ map onto a unique pair of $\mu_{a}$ and $\mu_{s}^{\prime }$ values [30], we hypothesize that $R_{T}$ and $slope_{logSRR}$ could be used as LUT’s inputs. The LUT is generated from a set of reflectometry and fluorescence measurements in phantoms – covering the range of optical properties of interest – with known fluorophore concentrations. In this way, the LUT also accounts for the optical characteristics of the illumination and imaging systems on the method parameters. By avoiding the calculation of explicit $\mu_{a}$ and $\mu_{s}^{\prime }$ and using analytical or numerical models, our method could minimize theoretical, numerical, and experimental uncertainty toward implementing a robust FMI correction.

We demonstrate the feasibility of the proposed method through MC-based simulations and the experimental retrieval of fluorophore concentrations in liquid and gel phantoms using an open-field imaging system. In these tests, we considered a wide range of $\mu_{a}$ and $\mu_{s}^{\prime }$ that can be found in biological tissues. Moreover, a near-infrared (NIR) fluorophore with short Stokes shift (Alexa Fluor 680) was used as its characteristics mirror the features of most of the currently used fluorescent agents for intra-operative fluorescence imaging (e.g., indocyanine green) [31–33]. The increased accuracy in these measurements supports the validity of our FI correction method. Finally, the potential clinical application of the developed methodology is discussed.

## 2. Methods

### 2.1 FI correction and quantification

The proposed method relies on creating a LUT to calculate a factor that minimizes the optical property dependence of FI ( F ). *F* was determined by remotely projecting a disk (size, 4.16 mm) on the sample and integrating the digitalized FI over the central area (diameter, 1.40 mm). The LUT inputs were two reflectometry quantities derived after processing the images of large (size, 4.16 mm) and small (size, 0.70 mm) disks projected on the sample. The first quantity was the total diffuse reflectance ( $R_{T}$ ). To calculate this, the large disk’s image was normalized with the wide-field image of a reflectance standard (e.g., Spectralon). Then, the intensities were integrated over a central area (diameter, 1.40 mm). To calculate the second quantity, the image of the small disk was processed to obtain the SRR or R(r) by angularly averaging the intensities as a function of the radial distance ( r ) from the beam center. Those illumination sizes parameters were empirically selected, getting a compromise between signal-to-noise ratios and spatial resolution when mapping a non-homogenous sample. Bigger sizes increase the signal-to-noise ratios, but decrease spatial resolution when mapping the sample. The sizes can be changed in further implementations of the method. The second LUT input corresponded to the slope of the $log_{10}[R(r)]$ (or $slope_{logSRR}$ ) between 1.1 and 2.1 mm away the beam center. This radial range was selected because the $log_{10}[R(r)]$ – across a wide range of optical properties – was well linearly fitted, as it was investigated numerically (see Sub-section 2.2). In addition, those radial positions are close to the beam, enhancing the signal-to-noise ratio when the intensity is recorded. $slope_{logSRR}$ is nearly proportional to $\mu_{eff}$ , as demonstrated in Supplement (Section 1). $R_{T}$ and $slope_{logSRR}$ were selected as LUT inputs because a unique pair of values ( $R_{T},$ $\mu_{eff}$ ) maps onto a unique pair of values ( $\mu_{a}$ , $\mu_{s}^{\prime }$ ) [30] and similarly, a pair of values ( $R_{T}$ , $slope_{logSRR}$ ) will map onto a correcting value for *F* under the effects of the tissue optical properties – represented by $\mu_{a}$ and $\mu_{s}^{\prime }$ – without requiring their estimation. The LUT output is the correcting value (or cvF ) to compute the optical property corrected FI ( $F_{corrected}$ ) as follows:

(1) $$F_{corrected}=F\cdot 10^{cvF(R_{T},slope_{logSRR})}$$

To create the LUT, *F*, $R_{T}$ and $slope_{logSRR}$ were measured in phantoms (or ‘training phantoms’) with the same geometry and fluorophore concentration ( $c_{cal}$ ), but with varying absorption and scattering properties within the range of interest according to the application. The LUT was created by interpolating cvF in the training dataset as follows:

(2) $$cvF(R_{T},slope_{logSRR})=log_{10}(1/F)$$

The logarithm and power of 10 in Eq. (1) and (2), respectively, were incorporated to minimize the variations on cvF when *F* was strongly attenuated.

Finally, the fluorophore concentration ( c ) in samples is given by:

(3) $$c=c_{cal}F_{corrected}$$

where the linear relationship between $F_{corrected}$ and *c* accounts for the properties of the fluorophore (extinction coefficient and quantum yield) but excludes the quenching effects.

### 2.2 Numerical calculations

F , $R_{T}$ and R(r) were numerically simulated to: i. verify the linearity of $log_{10}[R(r)]$ in the radial range where the $slope_{logSRR}$ was computed; ii. investigate the impact of the number of training phantoms on the accuracy of the corrected FI; iii. compare the performance of two types of interpolations when creating the LUT; and iv. compare the correction accuracy of our method and the standard F/R method.

A MC algorithm [34] was used to calculate the light interaction of a collimated, gaussian beam impinging a semi-infinite medium with a uniformly distributed fluorophore. Mimicking the experimental conditions, the programmed medium’s optical properties simulated liquid phantoms composed of: Intralipid (IL; Sigma-Aldrich Inc., USA) as scattering agent; Indian ink (Chartpak Inc., USA) as absorption agent; and Alexa Fluor 680 (Thermo Fisher Scientific, USA) as fluorophore. The programmed medium’s optical properties were representative of biological tissues [35]. While $\mu_{s}^{\prime }$ ranged from 2.5 to 100 cm^-1^, $\mu_{a}$ ranged from 0.05 to 3 cm^-1^ at the excitation wavelength (670 nm). $\mu_{a}$ and $\mu_{s}^{\prime }$ at the emission wavelength (716 nm) were estimated from the wavelength dependence of Intralipid [36] and the absorption spectra of Indian ink. The medium’s refractive index was set to 1.37.

The number of ‘training phantom’ was set to $N_{a}\times N_{s}$ , corresponding to the combinations of $N_{a}$ values for $\mu_{a}$ and $N_{s}$ values for $\mu_{s}^{\prime }$ . $\mu_{a}$ and $\mu_{s}^{\prime }$ were logarithmically spaced, covering their corresponding ranges. The total number of programmed $\mu_{a}$ and $\mu_{s}^{\prime }$ combinations ( $N_{total}$ ) was 900 resulting from maximum $N_{a}$ and $N_{s}$ of 20 and 25, respectively. For each $\mu_{a}$ and $\mu_{s}^{\prime }$ combination, 10^6^ photons were launched.

The geometric parameters for illumination (e.g., beam sizes) and detection (e.g., integration areas) to simulate *F*, $R_{T}$ , and SRR are specified in Section 2.1. The experimentally-derived reflectance from Spectralon [37] was also calculated and used to normalize $R_{T}$ . The SRR was normalized to the total energy and area around a disk (radius, 5 mm) centered at the incident position. The $slope_{logSRR}$ was calculated via linearly fitting the logSRR in the previously specified radial range. To generate the LUT, linear and thin-plate spline (TPS) interpolations were implemented in MATLAB (MathWorks, Natick, MA, USA).

The accuracy of the corrected FI across the entire simulated range of $\mu_{a}$ and $\mu_{s}^{\prime }$ was evaluated through the root-mean-square error (RMSE) between 1 (the expected $F_{corrected}$ , since all the programmed phantoms have the same fluorophore concentration) and $F_{corrected}$ . The RMSE was computed as:

(4) $$RMSE={\left[\frac{1}{N_{total}}\overset{N_{total}}{\underset{i=1}{\sum }}{\left(1-F_{corrected,i}\right)}^{2}\right]}^{1/2}$$

where *i* denotes each $\mu_{a}$ and $\mu_{s}^{\prime }$ combination.

### 2.3 Experimental validation

**Fig. 1.**
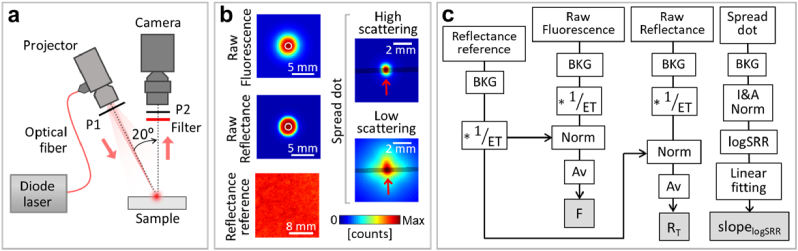
Acquisition and processing of measurements for FI correction. (a) Optical setup for the dynamic projection of the excitation light and the acquisition of reflectance and fluorescence. P1 and P2 are crossed linear polarizers. (b) Examples of raw images of fluorescence, reflectance, reference of reflectance, and a spread dot in a high or low scattering medium. (c) Quantification of *F*, $R_{T}$ and $slope_{logSRR}$ . After acquisition, all data shown in (b) are corrected for the background (BKG). The images of the fluorescence, reflectance, and reference of reflectance are normalized to the exposure time (*1/ET). Then, the reflectance and fluorescence are further normalized to the reflectance reference (Norm). *F* and $R_{T}$ correspond to the average (Av) of the values within the area depicted by the white circles in (b). On the other hand, the image of the spread dot is normalized to both the total intensity and area (I&A Norm) within a radius of 3.7 mm around the intensity peak. The radial profile of the logSRR is obtained from the intensity values across the shadowed strip in (b). The slope is calculated via linear fitting the logSRR between 1.1 and 2.1 mm.

To experimentally implement and test the proposed FI correction and quantification, we used the setup and procedure shown in Fig. 1. A digital light processing (DLP) projector (4500 Lightcrafter; Texas Instruments Inc., USA) focused light patterns at the surface of the sample, as depicted in Fig. 1(a). The optical engine of the projector was modified to use the light from a continuous wave fiber-coupled diode laser at 670 nm (BWF1-670-300E; B&W TEK Inc., USA). The light was conducted to the projector via a multimode fiber (M47L01; Thorlabs Inc., Germany). An eccentric rotating mass vibration motor (not shown in Fig. 1(a)) was attached to the fiber to reduce the speckle contrast of the illumination. The imaging system consisted of an achromatic doublet pair (MAP10100100-A1; Thorlabs Inc., Germany) and an electron-multiplying CCD camera (Luca; Andor, UK). The FI was acquired by placing a spectral filter (716 ± 40 nm; Edmund Optics, USA) in front of the imaging system. The mean intensity at the sample plane was 125 µW/cm^2^. The field of view of the system was 7.7 × 7.7 degrees^2^ or 35 × 35 mm^2^. Unlike the numerical simulations, the interrogating beam had an oblique incidence (20°) into the sample. Reflectance values were normalized using a diffuse reflectance standard (WS-1; Ocean Optics Inc., USA).

*F*, $R_{T}$ and $slope_{logSRR}$ were measured by projecting disks at desired locations onto the sample. The disk sizes are specified in Section 2.1. Figure 1(b) shows examples of raw acquired disks. The radial symmetry of the spread dot (i.e., the smallest disk) was reduced in low turbid media due to the oblique focusing of the rectangular projector’s matrix. Additionally, a reference reflectance image was acquired with the reflected intensity from a piece of white and thick paper located at the sample’s plane. The exposure times of the camera were: 10 s to acquire *F*; ranged from 0.15 to 0.8 s to acquire $R_{T}$ ; and ranged from 0.4 to 12 s to acquire the spread dots. Higher exposure times are required in samples with low scattering to increase the signal-to-noise ratio.

Figure 1(c) illustrates the processing of the acquired images to calculate $R_{T}$ , *F* and $slope_{logSRR}$ . The background was subtracted in all acquired images to minimize the effect of the camera’s dark noise and parasitic light. This background was defined as the digitalized intensity when a zero matrix was displayed on the projector. Reflectance and fluorescence images were first normalized to the exposure time. Spatial non-uniformities in the illumination were compensated by normalizing the reflectance and fluorescence images to the reflectance reference. $R_{T}$ and *F* are the average of the normalized images within the disk enclosed by the white circle in Fig. 1(b). On the other hand, the images of the spread dot were normalized to both the total intensity and area within a radius of 3.7 mm around the peak position. Because of the oblique incidence, the peak was slightly shifted in the direction of beam incidence (depicted by red arrows in Fig. 1(b)) with respect to the center of mass of the intensity distribution at larger distances. The profiles perpendicular to the direction of incidence, depicted by the shadowed strip in Fig. 1(b), were less sensitive to this effect. Therefore, the SRR was calculated by radially averaging the intensities within that strip (width, 0.46 mm). The slope was then calculated via linear fitting of the logSRR between 1.1 and 2.1 mm away from the peak position.

#### 2.3.1 Training phantoms

To generate the correcting LUT, we prepared 36 liquid phantoms, corresponding to $N_{a}$ =6 and $N_{s}$ =6. The phantoms consisted of solutions in distilled water of Intralipid, Indian ink, Alexa Fluor. The concentration of Alexa Fluor 680 (i.e., $c_{cal}$ ) was 87 nM for all phantoms. The concentrations of Intralipid and Indian ink were set to logarithmically distribute $\mu_{a}$ and $\mu_{s}^{\prime }$ calculated at 670 nm between [0.08, 2.57] cm^-1^ and [3.02, 96.8] cm^-1^, respectively. The characteristics, preparation and optical property calculation of each phantom are detailed in Supplement (Table S1). The solutions were contained in cylindrical wells of black polylactide with a diameter and depth of 11.6 and 10 mm, respectively. The disks were projected onto the wells’ center. The $slope_{logSRR}$ was calculated three times for different interrogating beams’ positions with lateral shifts of 0.14 mm. The LUT was fitted by TPS interpolation, using the mean of the three $slope_{logSRR}$ values per phantom.

#### 2.3.2 Liquid phantoms for testing

We first prepared 16 liquid phantoms with different fluorophore concentrations and optical properties, but the same geometry, to experimentally test the FI correction and quantification with the generated LUT. The $slope_{logSRR}$ was calculated three times, as was done previously for the training phantoms. From those calculations, three fluorophore concentrations were computed for each phantom. The characteristics, preparation and optical property calculation of each phantom are detailed in Supplement (Table S2).

To investigate the effect of the well depth on the FI correction, 16 additional phantoms were prepared by using four solutions with different optical properties but the same fluorophore concentration (87 nM). The solutions were contained in wells with depths of 1, 3, 5 and 10 mm. The characteristics, preparation and optical property calculation of each phantom are detailed in Supplement (Table S3).

#### 2.3.3 Gel phantoms for testing

To test the two-dimensional retrieval of fluorophore concentration, we prepared a gel phantom composed of three regions (R1, R2 and R3). The optical properties of R2 and R3 were similar, with higher scattering and lower absorption properties compared to those at R1. The nominal *c* at R3 was double of that in R1 and R2. The composition and preparation of the phantom is detailed in Supplement (Section 2.2). The phantom had a trapezoidal prism-shape with dimensions: 34.6 mm, base width and length; 27.9 mm, top width and length; and 8.0 mm, height. R2 and 3 were contained in two cylindrical wells with a diameter of 11 mm. $R_{T}$ , *F* and $slope_{logSRR}$ were measured sequentially, projecting the disks onto a grid with width and spacing of 18.6 mm and 1.84 mm, respectively. For the presentation of results, biharmonic spline interpolation was used to represent intermediate positions in the grid.

## 3. Results

### 3.1 FI correction and quantification: numerical proof-of-concept

**Fig. 2.**
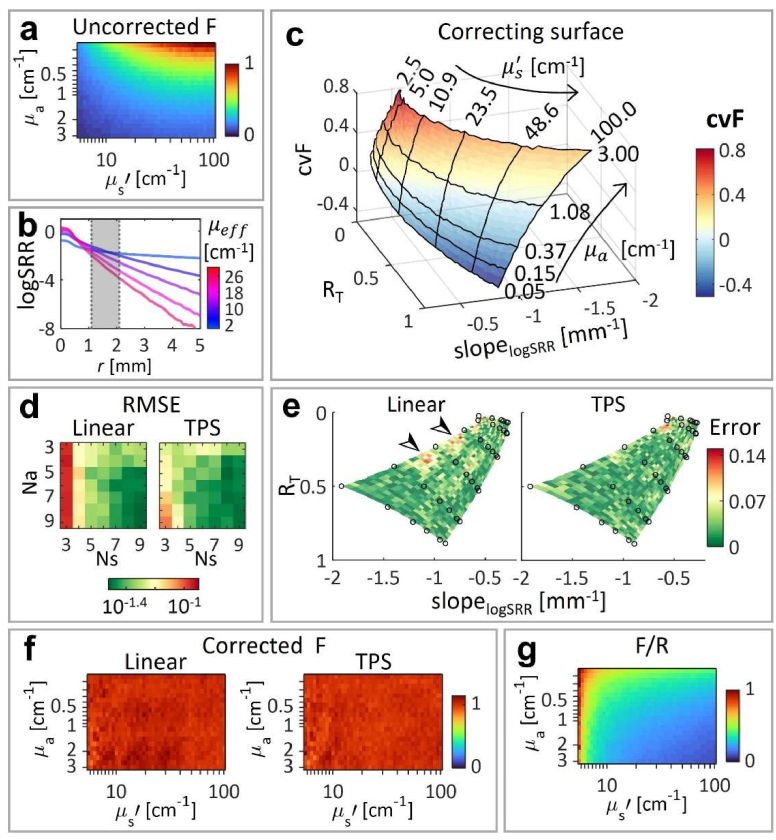
Fluorescence intensity (FI) correction with simulated quantities. (a) Normalized uncorrected FI as a function of $\mu_{a}$ and $\mu_{s}^{\prime }$ . (b) Examples of radial logSRR profiles, where the grey column marks the radial range for the slope calculation. (c) Dependence of the correcting values of FI ( cvF ) on $R_{T}$ and $slope_{logSRR}$ . The performance of the linear and thin-plate spline (TPS) interpolations were compared in terms of: (d) the root-mean-square error (RMSE) between the corrected FI and its expected value (i.e. 1) as a function of the number of the $\mu_{a}$ and $\mu_{s}^{\prime }$ values ( $N_{s}$ and $N_{a}$ , respectively); (e) the absolute difference between the corrected FI and 1 for $N_{s}$ and $N_{a}$ equal to 6, where the training quantities are depicted by black circles; and (f) the corrected FI as a function of $\mu_{a}$ and $\mu_{s}^{\prime }$ , for $N_{s}$ and $N_{a}$ equal to 6. (g) FI corrected by the standard F/R method as a function of the optical properties.

We first validated the FI correction method using simulated values as proof-of-concept. Figure 2(a) shows the dependence of the uncorrected *F* on $\mu_{a}$ and $\mu_{s}^{\prime }$ . Examples of the generated logSRR profiles are shown in Fig. 2(b) for several $\mu_{eff}$ values, where the shadowed region indicates the radial range over which the $slope_{logSRR}$ was calculated. Within this range, the mean coefficient of determination between the logSRR and the radius – across all the 900 $\mu_{a}$ and $\mu_{s}^{\prime }$ combinations – was 0.998 ± 0.002 (mean ± standard deviation), ranged from 0.984 to 1. Those high coefficients validated our hypothesis on the linearity of logSRR. Figure 2(c) depicts the correcting values cvF as a function of $R_{T}$ and $slope_{logSRR}$ simulated across all $\mu_{a}$ and $\mu_{s}^{\prime }$ combinations.

The FI was corrected using LUTs generated with several $N_{a}$ and $N_{s}$ values and two types of interpolations (linear and TPS). Figure 2(d) shows a RMSE comparison between the two interpolations as a function of $N_{a}$ and $N_{s}$ , indicating that TPS interpolation results in lower RMSE than linear interpolation. For $N_{a}$ =6 and $N_{s}$ =6, Fig. 2(e) shows a comparison of the absolute difference – or error – between 1 and $F_{corrected}$ obtained by the two interpolations. In both cases, the error is higher for lower absorption and higher scattering coefficients. Furthermore, in the case of the linear interpolation, the error is increased with higher $\mu_{a}$ values as marked by the arrows in Fig. 2(e). Figure 2(f) represents the previous $F_{corrected}$ values, with both interpolations, as a function of $\mu_{a}$ and $\mu_{s}^{\prime }$ . The mean relative standard deviations of those values are only 0.010 and 0.008 for the linear and TPS interpolations, respectively. Conversely, the standard F/R method leads to larger variations over the entire examined range of optical properties (Fig. 2 g). In summary, the fidelity of LUT-based FI correction was higher than the standard F/R method, especially when using the TPS interpolation.

### 3.2 Experimental validation

#### 3.2.1 Generation of the correcting LUT

**Fig. 3.**
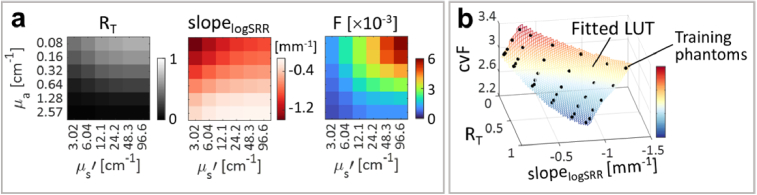
Generation of the correcting look-up table (LUT) by using liquid training phantoms. (a) Measurements to fit the LUT: total reflectance ( $R_{T}$ ), slope of the logSRR ( $slope_{logSRR}$ ), and fluorescence intensity ( F ). (b) Fitted LUT, with correcting values of the fluorescence intensity ( cvF ).

As the first step for the experimental demonstration of the LUT-based correction, the correcting LUT was generated by the measurements in the liquid training phantoms. Figure 3(a) shows the measured *F*, $R_{T}$ and $slope_{logSRR}$ as a function of the calculated $\mu_{a}$ and $\mu_{s}^{\prime }$ . The mean standard deviation of the $slope_{logSRR}$ after three measurements per phantom was 0.003 mm^-1^, corresponding to a coefficient of variation of 0.005. The correcting LUT was fitted via TPS interpolation of *F*, $R_{T}$ and the mean $slope_{logSRR}$ per phantom (see Fig. 3(b)).

#### 3.2.2 FI correction and quantification

**Fig. 4.**
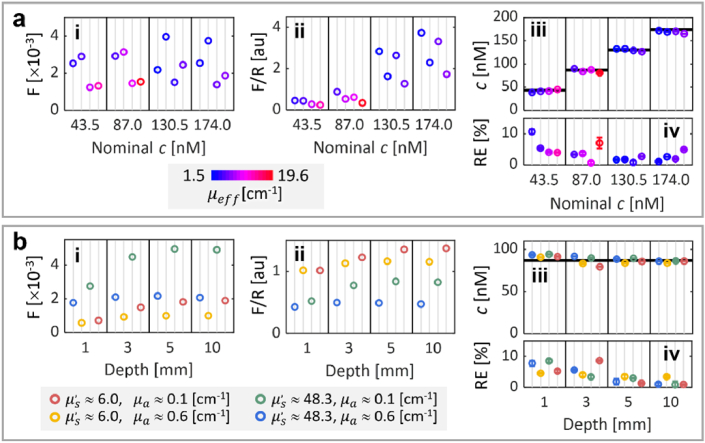
Experimental FI correction and quantification in liquid phantoms. (a) FI correction and quantification of different fluorophore concentrations ( c ) of Alexa Fluor 680: i. uncorrected fluorescence; ii. standard F/R correction; iii. *c* retrieved with the corrected FI by using the generated LUT; and iv. relative error (RE) between the nominal and retrieved *c*. (b) FI correction and quantification in liquid phantoms with different depths and optical properties, but the same c : i. uncorrected fluorescence; ii. standard F/R correction; iii. *c* retrieved with the corrected FI by using the generated LUT; and iv. relative error (RE) between the nominal and retrieved *c*. Data are represented as the mean ± standard deviation for each quantity in (a.iii), (a.iv), (b.iii), and (b.iv), after measuring the $slope_{logSRR}$ three times for each phantom. Horizontal black bars in (a.iii) and (b.iii) represent the nominal *c*. au, arbitrary units.

Figure 4 shows the experimental FI correction and quantification in liquid phantoms. First, the correction was applied to phantoms with the same geometry but different optical properties and fluorophore concentrations, as depicted in Fig. 4(a). The processing of the uncorrected FI (Fig. 4(a).i) by the standard F/R method did not accurately account for *c*, as seen from the large data variations shown in Fig. 4(a).ii. Conversely, *c* was accurately retrieved using the FI corrected by the generated LUT (in Fig. 3(b)), as shown in Fig. 4(a).iii where horizontal black lines depict the nominal *c*. The coefficient of variation of the retrieved *c* over all phantoms was 0.004. Figure 4(a).iv shows the relative error (RE) between the nominal and retrieved *c*, with an overall RE of 3.54 ± 0.12% (mean ± standard deviation).

Second, the correction was applied in phantoms with different depths but the same nominal *c*, as depicted in Fig. 4(b). The uncorrected *F* (Fig. 4(b).i) and standard F/R correction (Fig. 4(b).ii) were more dependent on depth in phantoms with lower absorption, and standard F/R correction did not compensate for the variations in scattering and absorption properties. The LUT-based FI correction allowed accurate retrieval of *c*, as shown in Fig. 4(b).iii. The coefficient of variation of the retrieved *c* over all phantoms was 0.005. In general, the RE (Fig. 4(b).iv) was seen to decrease as the depth approached 10 mm (the depth used to generate the correcting LUT), with an overall mean RE of 3.93 ± 0.13%.

#### 3.2.3 Two-dimensional FI quantification

**Fig. 5.**
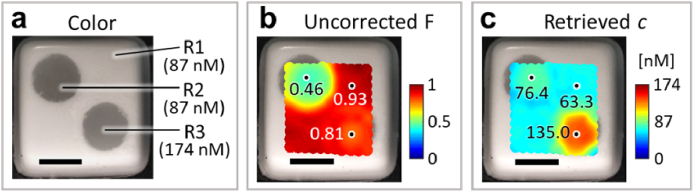
Two-dimensional FI correction and quantification in a gel phantom. (a) Color image of the phantom depicting three regions (R1, R2 and R3) and their nominal concentrations of Alexa Fluor 680. (b) Interpolated measured fluorescence intensities overlaid on the color image. (c) Interpolated retrieved fluorophore concentrations overlaid on the color image. Values at the marked positions in (b) and (c) correspond to the average of each quantity within a square with a side length of 3.7 mm. Scale bar, 10 mm.

Figure 5(a) shows the gel phantom and its nominal fluorophore concentrations. In general, the normalized *F* from the point-scanning measurements (Fig. 5(b)) does not match the relative concentration of the phantom’s regions. For example, according to the average *F* values around the positions marked in Fig. 5(b), the FI at R3 is 0.87- and 1.76-fold the FI at R1 and R2 (rather than 2-fold), respectively, corresponding to REs of 56.5% and 12.0% with respect to the ratio of nominal concentrations. Figure 5(c) shows the retrieved *c* values after applying the LUT-based FI correction, which are lower than the nominal *c* values. However, the retrieved values preserve the relative nominal concentrations among the regions. According to the average concentrations around the positions marked in Fig. 5(c), the *c* retrieved at R3 is 2.13- and 1.77–fold higher than at R1 and R2, respectively, corresponding to REs of 6.6% and 11.6% with respect to the nominal ratio.

## 4. Discussion

We have developed a simple and model-independent method for correcting the dependence of FI on the optical properties ( $\mu_{a}$ and $\mu_{s}^{\prime }$ ) of tissue-like media. The method is functional over a wide range of $\mu_{a}$ and $\mu_{s}^{\prime }$ , and its major advantage is the lack of a comparison between measurements and numerical (or analytically calculated) quantities. The processing needed for FI correction and quantification is simplified by using a LUT, generated from measurements in phantoms with the same geometry and fluorophore concentration. The LUT calculates a factor to correct FI according to the measured total diffuse reflectance and the slope of the logSRR. This method proved to be higher in accuracy compared to the standard F/R correction in both numerical and experimental tests.

Our method was experimentally validated by retrieving the concentrations of Alexa Fluor 680 in phantoms with different optical properties. The mean relative errors between the retrieved and nominal *c* were 3.54% in liquid phantoms with different *c* but the same geometry, and 3.93% in liquid phantoms with the same *c* but different depths. Those errors are lower than those previously reported with a similar NIR, short Stokes fluorophore. For example, Valdes *et al.* [23] reported a mean relative error of 4.8% in liquid phantoms with ZW800-1 as fluorophore and a narrower ranges of optical properties that the considered ones in this paper. This approach required to estimate $\mu_{a}$ at the excitation wavelength and measuring the reflectance at the emission and excitation wavelengths, increasing the complexity of both system configuration and processing with respect to our method.

We also retrieved the relative concentration of Alexa Fluor 680 in different spatial regions of a gel phantom. The error of the relative *c* between two regions with different optical properties (R1 and R2) was reduced by 88% when using FIs corrected with LUT compared to using non-corrected FIs. In regions with similar optical properties (R2 and R3), the error in relative *c* (11.6%) was a direct consequence of error in the non-corrected FI (12%). The average relative error between the nominal and retrieved *c* was approximately 21%. These errors were likely due to the changes in Alexa Fluor 680 quantum yield resulting from the high temperatures during phantom preparation [38].

It is necessary to point out that our method calculates the correcting value for the FI from measurements taken only at the excitation wavelength. This approach is supported by the fact that the tissues’ optical properties are less dependent on the wavelength in the NIR than the visible range. It is known that in soft tissues, where $\mu_{s}^{\prime }$ monotonically decreases with the wavelength, $\mu_{a}$ is related to the molar extinction of oxy-hemoglobin [39], which has a coefficient of variation that is 0.30 times lower between 650-1000 nm than between 380-650 nm. Other previously reported methods for FI correction [7,8,18,26,27] incorporate the reflectance and/or optical properties ( $\mu_{a}$ and $\mu_{s}^{\prime }$ ) at the emission wavelength. Such methods were developed and applied in experiments with fluorophores that have larger Stokes shift and excitation wavelength in the ultra-violet or visible spectrum (e.g., 5-ALA-PpIX). The accuracy of our method for fluorescent agents with shorter excitation wavelengths and larger Stokes shifts has not been tested here and further investigation is needed in the future.

A DLP-based open-field imaging system was used to experimentally demonstrate the increased accuracy offered by our method. Regarding this system, it is necessary to point out that the exposure times of the camera can be reduced using hardware with better performance (i.e., a more powerful laser, a higher sensitive camera, and a DLP operating at much higher frame rates than 60 Hz – the current one –). In this configuration, wide-field fluorescence image is generated after raster scanning the sample which can slow the acquisition process and limit the spatial resolution. One possible solution to increase the spatial resolution is to reduce the size of the disks to measure $R_{T}$ and *F*, but this approach requires increasing the exposures times (i.e., to slow the scanning). Therefore, to speed up the acquisition without compromising spatial resolution, we are conducting further tests to adapt our LUT-based correction to spatial frequency domain imaging. In this technique, the diffuse reflectance at zero and nonzero spatial frequencies, currently used to estimate $\mu_{a}$ and $\mu_{s}^{\prime }$ , would be the input parameters for the LUT used for correction.

We have identified the following sources contributing to error in the test of our method: i. the fit of the correcting LUT, which led to relative errors of up to 14.64% with a mean of 2.62% across all $\mu_{a}$ and $\mu_{s}^{\prime }$ combinations, according to the numerical calculations for $N_{a}$ =6 and $N_{s}$ =6 (Fig. 2(e)); ii. the temporal fluctuations of the illumination intensity (up to 6% in a measurement session), which were uncompensated; and iii. the effect of the surface roughness in the gel phantom. It is known that the surface roughness affects slope-based optical property estimation [40–42], and its impact on our method needs to be further studied.

Finally, it is important to mention that our FI correction method could also be implemented in other fluorescence systems such as point-probes or endoscopes. The radial range where the $slope_{logSRR}$ was calculated (1.1 to 2.1 mm from the source) is a possible advantage for these implementations in the future. In this way, our method could allow for the standardization of different types of fluorescence systems.

In conclusion, we have developed a model-independent method for accurate FI determination with optical property correction. By generating and applying a LUT based on the total reflectance and spatial variation of the reflectance of training phantoms, our method can successfully retrieve accurate fluorophore concentrations from samples with a wide range of optical properties and varying depths. This novel method could therefore be a significant step towards the standardization of fluorescence-based systems in clinical practice and the improvement of outcomes after image-guided interventions (e.g., tumor resection).

## Data Availability

Data underlying the results presented in this paper are not publicly available at this time but may be obtained from the authors upon reasonable request.

## Supplemental document

See Supplement 1 for supporting content.
