# Supplementary material for: Using reflectometry to minimize the dependence of fluorescence intensity on optical absorption and scattering
Source: Biomed Opt Express. 2023 Sep 27;14(10):5499–511. doi: 10.1364/BOE.496599 (PMC10581795; doi:10.1364/BOE.496599)
Supplement: Supplementary file 1 [file boe-14-10-5499-s001.pdf]

Supplemental Document

Biomedical Optics EXPRESS

Using reflectometry to minimize the dependence of fluorescence intensity on optical absorption and scattering: supplement

AUGUSTO ARIAS,[1,2] MARIA ANASTASOPOULOU,[1,2] DIMITRIS GORPAS,[1,2] AND VASILIS NTZIACHRISTOS[1,2,3,*]

[1] *Chair of Biological Imaging at the Central Institute for Translational Cancer Research (TranslaTUM), School of Medicine, Technical University of Munich, Munich, 81675, Germany*
[2] *Institute of Biological and Medical Imaging, Helmholtz Zentrum München, Neuherberg, 85764, Germany*
[3] *DZHK (German Centre for Cardiovascular Research), partner site Munich Heart Alliance, Munich, 81675, Germany*
[*] *bioimaging.translatum@tum.de*

This supplement published with Optica Publishing Group on 27 September 2023 by The Authors under the terms of the Creative Commons Attribution 4.0 License in the format provided by the authors and unedited. Further distribution of this work must maintain attribution to the author(s) and the published article's title, journal citation, and DOI.

Supplement DOI: https://doi.org/10.6084/m9.figshare.24126987

Parent Article DOI: https://doi.org/10.1364/BOE.496599

USING REFLECTOMETRY TO MINIMIZE THE DEPENDENCE OF FLUORESCENCE INTENSITY ON OPTICAL ABSORPTION AND SCATTERING

The Supplemental Material is organized as follows: In Sec. 1, we show the relationship between the effective attenuation coefficient (μ_{eff}) and the slope of the logarithmic values of the spatially resolved reflectance. In Sec. II, we describe the preparation, characteristics and optical property calculation of the phantoms used for generating and testing of the look-up table (LUT) used to correct fluorescence intensities (FIs).

1. Relationship between μ_{eff} and the slope of the logarithmic spatially resolved reflectance

We determine and compare the relationship between μ_{eff} and the slope of the logarithmic values of the spatially resolved reflectance (SRR or $R(r)$, where r is the radial distance from the beam position on the sample surface), as adapted in our work (i.e., $log_{10}[R(r)]$), and following the diffusion approximation-based formulation of Patterson *et al.* [1] (i.e., $log_{10}[r^2R(r)]$).

First, we calculated the SRR from a pencil beam impinging on a semi-infinite medium (refractive index, n_m=1.4) – with μ_a and μ_s' coefficients – from air. The SRR is modelled by the diffusion theory as in [2]:

$$R(r) = \frac{\mu_s'}{4\pi(\mu_t')^2}\left[\left(\mu_{eff} + \frac{1}{r_1}\right)\frac{\exp\left(-\mu_{eff}r_1\right)}{{r_1}^2} + \left(1+\frac{4}{3}A\right)\left(\mu_{eff} + \frac{1}{r_2}\right)\frac{\exp\left(-\mu_{eff}r_2\right)}{{r_2}^2}\right] \quad (S.1)$$

where:

$$r_1 = \sqrt{\left(\frac{1}{\mu_t'}\right)^2 + r^2},\ r_2 = \sqrt{\left[\frac{1}{\mu_t'}\left(1+\frac{4}{3}A\right)\right]^2 + r^2},$$

$$A = \frac{-1.440n_m^{-2}+0.710n_m^{-1}+0.0636n_m+1.668}{+1.440n_m^{-2}-0.710n_m^{-1}-0.0636n_m+0.332}$$

and $\mu_t' = \mu_a + \mu_s'$. Then, the slope of $log_{10}[r^2R(r)]$ and $log_{10}[R(r)]$ were calculated for r between 3 and 4 mm. Those radii are within the diffusion approximation (i.e., $r \gg 1/\mu_t'$).

Figure S1 shows the results of the calculations and analysis. The comparison of $log_{10}[r^2R(r)]$ and $log_{10}[R(r)]$ for high and low scattering mediums is shown in Figure S1a. Unlike the slope of $log_{10}[R(r)]$, the slope of $log_{10}[r^2R(r)]$ is not always negative. The radial position of the stationary point of $log_{10}[r^2R(r)]$ depends on μ_t'. Despite such differences, the slope of $log_{10}[r^2R(r)]$ and $log_{10}[R(r)]$ equally accounts for μ_{eff}, as shown in Figure S1b. Both slopes decrease as μ_{eff} increases.

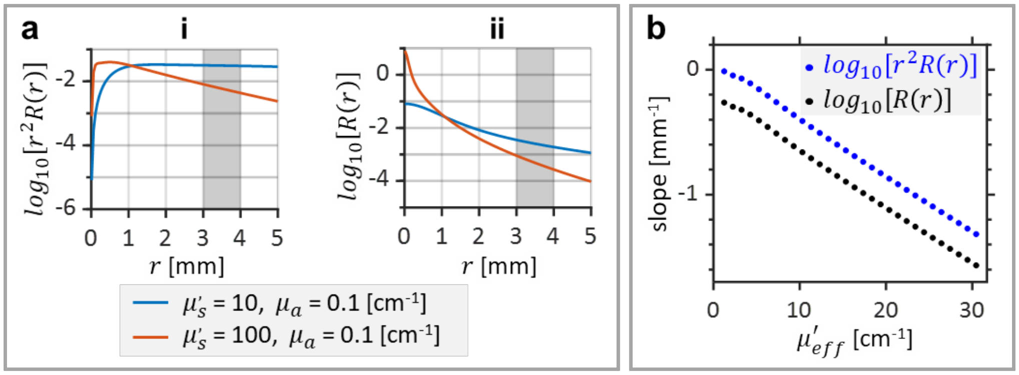

Fig. S1. Relationship between the spatial variation of the SRR and μ_{eff}. (a) Radial dependence of: i. $log_{10}[r^2R(r)]$ and ii. $log_{10}[R(r)]$. (b) Slope of $log_{10}[r^2R(r)]$ and $log_{10}[R(r)]$, calculated in the radial range shadowed in (a), as a function of the μ_{eff}.

2. Preparation, characteristics and optical property calculation of the used phantoms

2.1 Liquid phantoms

Training phantoms

To prepare the training phantoms, Indian ink was initially dissolved in distilled water to prepare a 'dark' buffer solution. This solution was serially diluted to prepare six solutions with logarithmically distributed optical densities (OD) at 670 nm as follows: 0.072, 0.144, 0.286, 0.572, 1.146 and 2.300. The ODs were measured using a spectrometer (USB4000 FL; Ocean Optics Inc., USA) in a transmission setup. μ_a was calculated as a function of OD by applying Beer-Lambert's law [3]:

$$\mu_a(OD) = log[10]\frac{OD}{\ell} \qquad \text{(S.2)}$$

where $log[\,]$ is the natural logarithm operation and ℓ is the cuvette thickness for the measurements of absorbance. In our experience, $\ell \equiv 1.03$ cm.

On the other hand, a 'scattering' buffer solution of Intralipid (IL) at 16% was prepared and serially diluted to obtain six solutions with IL percentages ($\%IL$) of: 16, 8, 4, 2, 1 and 0.5. μ_s' of these solutions were calculated by using Staveren's formula [4], assuming a linear relationship between this coefficient and $\%IL$:

$$\mu_s'(\%IL) = [160\lambda^{-2.4}(-0.1 + 0.58\lambda)]\frac{\%IL}{10} \quad [\text{cm}^{-1}] \qquad \text{(S.3)}$$

where λ is the wavelength in microns (i.e., $\lambda \equiv 0.670$ µm).

Finally, 36 training phantoms were prepared by mixing equal parts – by weight – of the dark and scattering solutions. Alexa Fluor 680 was added according to the weight of the mixture. The characteristics of the training phantoms are listed in Table S1. μ_a and μ_s' of the phantoms were estimated to half of those coefficients calculated in the stock solutions, when ignoring the scattering of the Indian ink, the absorption of the IL, and the optical properties of the Alexa Fluor 680.

Table S1. Characteristics of the training phantoms. c, concentration of Alexa Fluor 680.

	OD	% IL	μ_a [cm^{-1}]	μ_s' [cm^{-1}]	c [nM]	Depth [mm]
1	0.036	0.25	0.08	3.02	87.0	10
2	0.036	0.50	0.08	6.04	87.0	10
3	0.036	1.00	0.08	12.07	87.0	10
4	0.036	2.00	0.08	24.15	87.0	10
5	0.036	4.00	0.08	48.30	87.0	10
6	0.036	8.00	0.08	96.59	87.0	10
7	0.072	0.25	0.16	3.02	87.0	10
8	0.072	0.50	0.16	6.04	87.0	10
9	0.072	1.00	0.16	12.07	87.0	10
10	0.072	2.00	0.16	24.15	87.0	10
11	0.072	4.00	0.16	48.30	87.0	10
12	0.072	8.00	0.16	96.59	87.0	10
13	0.143	0.25	0.32	3.02	87.0	10
14	0.143	0.50	0.32	6.04	87.0	10
15	0.143	1.00	0.32	12.07	87.0	10
16	0.143	2.00	0.32	24.15	87.0	10
17	0.143	4.00	0.32	48.30	87.0	10
18	0.143	8.00	0.32	96.59	87.0	10
19	0.286	0.25	0.64	3.02	87.0	10
20	0.286	0.50	0.64	6.04	87.0	10
21	0.286	1.00	0.64	12.07	87.0	10
22	0.286	2.00	0.64	24.15	87.0	10
23	0.286	4.00	0.64	48.30	87.0	10
24	0.286	8.00	0.64	96.59	87.0	10
25	0.573	0.25	1.28	3.02	87.0	10
26	0.573	0.50	1.28	6.04	87.0	10
27	0.573	1.00	1.28	12.07	87.0	10
28	0.573	2.00	1.28	24.15	87.0	10
29	0.573	4.00	1.28	48.30	87.0	10
30	0.573	8.00	1.28	96.59	87.0	10
31	1.150	0.25	2.57	3.02	87.0	10
32	1.150	0.50	2.57	6.04	87.0	10
33	1.150	1.00	2.57	12.07	87.0	10
34	1.150	2.00	2.57	24.15	87.0	10
35	1.150	4.00	2.57	48.30	87.0	10
36	1.150	8.00	2.57	96.59	87.0	10

To test the LUT

The phantoms' preparation followed the procedure described above for the training phantoms. In the first experiment, the fluorescence intensity correction was tested in phantoms with the same geometry but different concentrations of Alexa Fluor 680 (c). Six and seven aqueous stock solutions of Indian ink and IL, respectively, were prepared with an approximated logarithmic distribution of OD and $\%IL$. Those solutions were mixed in equal parts by weight. Then, the Alexa Fluor 680 was added according to the weight of the mixture. The characteristics of the phantoms are listed in Table S2. μ_a and μ_s' were estimated as described before.

Table S2. Characteristics of the phantoms with the same geometry but different fluorophore concentration.

	OD	% IL	μ_a [cm^{-1}]	μ_s' [cm^{-1}]	c [nM]	Depth [mm]
1	0.048	3.00	0.11	36.22	43.5	10
2	0.048	6.00	0.11	72.44	43.5	10
3	0.383	3.00	0.86	36.22	43.5	10
4	0.383	6.00	0.86	72.44	43.5	10
5	0.192	1.50	0.43	18.11	87.0	10
6	0.192	6.00	0.43	72.44	87.0	10
7	0.767	1.50	1.71	18.11	87.0	10
8	0.767	6.00	1.71	72.44	87.0	10
9	0.072	0.38	0.16	4.53	130.5	10
10	0.072	1.00	0.16	12.07	130.5	10
11	0.383	0.38	0.86	4.53	130.5	10
12	0.383	1.00	0.86	12.07	130.5	10
13	0.144	0.38	0.32	4.53	174.0	10
14	0.144	0.75	0.32	9.06	174.0	10
15	0.767	0.38	1.71	4.53	174.0	10
16	0.767	0.75	1.71	9.06	174.0	10

In the second experiment, the fluorescence intensity correction was tested in phantoms with the same concentrations of Alexa Fluor 680 but different depths. Two aqueous stock solutions of Indian Ink and IL were prepared, by mixing them in equal parts – by weight – and adding the Alexa Fluor 680 according to the weight of the mixture. The characteristics of the phantoms are listed in Table S3. μ_a and μ_s' were estimated as described before.

2.2 Gel phantom

The gel phantom, composed of three regions, was prepared from three solutions by volume (one per phantom region). The compositions of the solutions are detailed in Table S4. The preparation of each solution was started by dissolving 450 mg of agar (Noble Agar; US Biological, United States) in water at 90°C (3.2°C above the melting point) using a magnetic stirring bar. IL and Indian ink were added while the temperature was reduced to 40°C (5.6°C above the gel point). Then, the Alexa Fluor 680 was added according to the weight of the solution. The mix was stirred while the temperature decreased, then transferred to the mold. μ_a was roughly estimated as 0.10 cm^{-1} for solution 1, and 0.41 cm^{-1} for solutions 2 and 3. On the other hand, μ_s' cannot be estimated by Equation S.3 due to the dependence of the scattering properties of IL on the agar content [5] and the mixing temperature and time [6].

Table S3. Characteristics of the phantoms with the same fluorophore concentration but different depths.

	OD	% IL	μ_a [cm^{-1}]	μ_s' [cm^{-1}]	c [nM]	Depth [mm]
1	0.036	0.50	0.08	6.04	87.0	1
2	0.286	0.50	0.64	6.04	87.0	1
3	0.036	4.00	0.08	48.30	87.0	1
4	0.286	4.00	0.64	48.30	87.0	1
5	0.036	0.50	0.08	6.04	87.0	3
6	0.286	0.50	0.64	6.04	87.0	3
7	0.036	4.00	0.08	48.30	87.0	3
8	0.286	4.00	0.64	48.30	87.0	3
9	0.036	0.50	0.08	6.04	87.0	5
10	0.286	0.50	0.64	6.04	87.0	5
11	0.036	4.00	0.08	48.30	87.0	5
12	0.286	4.00	0.64	48.30	87.0	5
13	0.036	0.50	0.08	6.04	87.0	10
14	0.286	0.50	0.64	6.04	87.0	10
15	0.036	4.00	0.08	48.30	87.0	10
16	0.286	4.00	0.64	48.30	87.0	10

Table S4. Composition of solutions used in the preparation of the gel-based phantom.

	Solution		
Components	1	2	3
16% IL [mL]	1.50	0.28	0.28
Indian ink (OD of 1.150 at 670 nm) [mL]	0.36	1.44	1.44
Water [mL]	7.14	7.28	7.28
Alexa Fluor 680 [nM]	87	87	174

References

1. M. S. Patterson, E. Schwartz, and B. C. Wilson, "Quantitative Reflectance Spectrophotometry For The Noninvasive Measurement Of Photosensitizer Concentration In Tissue During Photodynamic Therapy," in *Photodynamic Therapy: Mechanisms* (SPIE, 1989), Vol. 1065, p. 115.
2. T. J. Farrell, M. S. Patterson, and B. Wilson, "A diffusion theory model of spatially resolved, steady-state diffuse reflectance for the noninvasive determination of tissue optical properties in vivo," Med. Phys. **19**, 879–888 (1992).
3. B. C. Wilson and S. L. Jacques, "Optical Reflectance and Transmittance of Tissues: Principles and Applications," IEEE J. Quantum Electron. **26**, 2186–2199 (1990).
4. H. J. van Staveren, C. J. M. Moes, J. van Marie, S. A. Prahl, and M. J. C. van Gemert, "Light scattering in lntralipid-10% in the wavelength range of 400–1100 nm," Appl. Opt. **30**, 4507 (1991).
5. R. Cubeddu, A. Pifferi, P. Taroni, A. Torricelli, and G. Valentini, "A solid tissue phantom for photon migration studies," Phys. Med. Biol. **42**, 1971 (1997).
6. P. Lai, X. Xu, and L. V. Wang, "Dependence of optical scattering from Intralipid in gelatin-gel based tissue-mimicking phantoms on mixing temperature and time," J. Biomed. Opt. **19**, 035002 (2014).
